# Identification of HLA-A2-Restricted Mycobacterial Lipoprotein Z Peptides Recognized by T CellsFrom Patients With ActiveTuberculosis Infection

**DOI:** 10.3389/fmicb.2018.03131

**Published:** 2018-12-21

**Authors:** Yuan-yong Liu, Wei Sha, Shiqiang Xu, Xu-wei Gui, Liliang Xia, Ping Ji, Shujun Wang, Guo-ping Zhao, Xiao Zhang, Yingying Chen, Ying Wang

**Affiliations:** ^1^School of Life Science and Technology, Changchun University of Science and Technology, Changchun, China; ^2^Department of Microbiology and Immunology, Shanghai Institute of Immunology, Shanghai Jiao Tong University School of Medicine, Shanghai, China; ^3^Clinic and Research Center of Tuberculosis, Shanghai Key Laboratory of Tuberculosis, Shanghai Pulmonary Hospital, Tongji University School of Medicine, Shanghai, China; ^4^Shanghai-MOST Key Laboratory of Health and Disease Genomics, Chinese National Human Genome Center at Shanghai, Shanghai, China

**Keywords:** tuberculosis, mycobacterial lipoprotein Z, HLA-A2-restricted peptide, antigenicity, CD8^+^ and CD4^+^ T cells responses

## Abstract

Identification of HLA-restricted peptides derived from mycobacterial antigens that are endowed with high affinity and strong antigenicity is not only of interest in tuberculosis (TB) diagnostics and treatment efficacy evaluation, but might also provide potential candidates for the development of therapeutic vaccines against drug-resistant TB. Our previous work demonstrated that lipoprotein Z (LppZ) displayed high immunogenicity and antigenicity in active TB patients. In the present study, ten HLA-A2-restricted LppZ peptides (LppZp1-10) were predicted by bioinformatics, among which LppZp7 and LppZp10 were verified to possess high affinity to HLA-A2 molecules using T2 cell-based affinity binding assay. Moreover, results from ELISpot assay showed that both LppZp7 and LppZp10 peptides were able to induce more IFN-γ producing cells upon *ex vivo* stimulation of PBMC from HLA-A2^+^ active TB (ATB) patients as compared to those from healthy controls (HCs). Also, the numbers of LppZp7 and LppZp10-specific IFN-γ producing cells exhibited positive correlations with those of ESAT-6 peptide (E6p) or CFP-10 peptide (C10p) in ATB. Interestingly, stimulation with LppZp7/p10 mixture was able to induce higher intracellular expression of IFN-γ and IL-2 cytokines in CD8^+^ and CD4^+^ T cells from ATB as compared to HC, associated with lower expression of TNF-α in both CD8^+^ and CD4^+^ T cells. Taken together, HLA-A2-restricted LppZp7 and LppZp10 peptides display high immunoreactivity in HLA-matched ATB patients demonstrated by high responsiveness in both CD8^+^ and CD4^+^ T cells. With the ability to induce strong antigen-specific cellular responses, LppZp7 and LppZp10 are of potential value for the future applications in the prevention and control of TB.

## Introduction

Tuberculosis (TB) is the leading cause of death from the single infectious agent, *Mycobacterium tuberculosis* (*M.tb*). It is still one of the most severe global health problems. In 2016, there were an estimated 10.4 million newly onset TB patients and 1.04 million death (WHO, 2017). The number of new TB cases in China was nearly 0.9 million, ranking fourth worldwide after India, Indonesia, and Nigeria (WHO, 2017). The complexity of its pathogenesis and pathology makes the prevention and control of TB more challenging. For instance, accurate and rapid diagnosis for TB is still insufficient. The rapid increase of multiple drug-resistant (MDR) and extensively drug-resistant (XDR) TB in clinic leads to the longer duration of the treatment and the use of more expensive drugs whereas less efficacy, increasing the economic burden as well as the morbidity of global TB (Basak et al., 2016). Bacille Calmette-Guérin (BCG) is the only vaccine available for the prevention of TB, which is widely used in high-incidence regions. But its protection is demonstrated to be limited between 5 and 10 years (Cernuschi et al., 2018). Therefore, to develop new vaccines either for the prevention of TB or the immunotherapy of drug-resistant TB in the future is worthy of exploration.

T lymphocytes are considered to be the main cellular component to exert protection against *M.tb*. Unlike macrophages possessing phagocytosis ability through the direct clearance of bacilli (Sartain et al., 2006), T cells eliminate *M.tb* largely by lysing infected cells (Flynn and Chan, 2001). Both CD8^+^ and CD4^+^ T cells are involved in the immune protection offered by TB vaccine (Li et al., 2009). They initiate cellular immune responses through recognizing antigenic peptides presented by HLA class I and II molecules on host antigen presenting cells, respectively. Compared to the whole antigens, antigenic peptides have more advantages in the preparation and commercial applications, such as high purity in synthesis and containing less harmful antigenic components (Mollica et al., 2013). Early secretary antigenic target protein 6 (ESAT-6) and culture filtrate protein 10 (CFP-10) are known as two immunodominant antigens derived from *M.tb*. The artificially synthesized antigenic peptide pools are used in *M.tb* antigen-specific IGRA assay, including T.SPOT^®^.TB and QFT^®^ for the immune diagnosis of TB, which can avoid the interference of endogenous endotoxin existing in the purified proteins. However, these two antigens are still not good enough in neither TB diagnosis (Hemmati et al., 2016) nor vaccine development (Jeon et al., 2013). Gao et al. (2009) has recently reported that HLA-restricted mycobacterial peptides-based DNA vaccine could potentially trigger increased Th1 immune response in a mouse model. A CFP21-derived HLA-A2-restricted epitope could enhance the activity of cytotoxic T lymphocytes (Lv et al., 2010). However, only fusion protein containing CFP21 could enhance the protection against *M.tb* infection (Wang et al., 2011). Hence, to screen new mycobacterial antigenic peptides with high affinity and strong antigenicity is still in urgent need. Among all HLA-A alleles, HLA-A2 is the most common subtype in Asian population with an estimated frequency of more than 40% (Mehra et al., 2001). Therefore, identification of HLA-A2-restricted epitopes derived from *M.tb* proteins is of large application in the future.

Mycobacterial lipoprotein Z (LppZ), encoded by *rv3006*, is a newly identified antigens with good antigenicity in active TB patients (Xiao et al., 2016). It is a conserved lipoprotein with enzymes and metabolic activities in various mycobacterial strains (Sutcliffe and Harrington, 2004), including *M.bovis* and *M.tb*. Being one of the most immunogenic proteins with high antibody-to-protein ratio among culture filtrate proteins (Malen et al., 2008), it is reported to be sero-reactive in both cavitary and non-cavitary TB patients with sero-diagnostic potential for TB (Sartain et al., 2006). Our previous work has also reported that LppZ-specific IgA levels in TB patients and latent TB infection (LTBI) individuals were dramatically higher than that in healthy controls (HCs). LppZ-specific IgA level decreased substantially along with the anti-TB treatment (Xiao et al., 2017). What’s more, its potential as a new TB vaccine candidate antigen was evaluated with enhanced immune protection against *M.tb* challenge in mouse models (under revision manuscript). In the present study, potential HLA-A2 epitopes derived from LppZ were predicted by bioinformatic tools. Their immunoreactivity in HLA-A2^+^ active TB patients were further determined. Our study thus intended to provide key evidence for the construction of peptide-based TB vaccine and diagnostic approach.

## Materials and Methods

### Study Populations

Active tuberculosis (ATB) patients were included in the study. All ATB patients were in-patients from Shanghai Pulmonary Hospital affiliated to Tongji University School of Medicine, and were confirmed based on medical history, chest radiograph (X-ray and CT), acid-fast bacilli (AFB) smear or sputum culture. All the patients were both HIV- and HBV-negative. They have signed voluntary informed consent before being enrolled in this study. Both HCs and LTBIs were from healthy blood donors undergoing annual physical examination in Ruijin Hospital (Shanghai, China). They had no medical history or disease symptoms. LTBI subjects were detected by ESAT-6 or CFP-10 induced IFN-γ releasing cells, and were defined as more than 6 SFUs. Those subjects were eliminated from this study. All individuals involved in this study were adults who had been vaccinated with BCG Shanghai strain (Shanghai Institute of Biological Products Co., Ltd., Shanghai, China) during childhood. This study was approved by the Ethical Committee of Shanghai Jiao Tong University School of Medicine.

### Peptide Synthesis

The HLA-A2-restricted LppZ peptides were predicted on the website http://www-bimas.cit.nih.gov/molbio/hla_bind. ESAT-6 peptide mixture (E6p), CFP-10 peptide mixture (C10p) (Yang et al., 2017), HLA-A2-restricted LppZ peptides (LppZp1-10) and HLA-A2-restricted OVA66 peptide L235 (sequence FLPDHINIV) (Jin et al., 2005) were synthesized by Sangon Biotech (Shanghai, China). The peptides were purified by high performance liquid chromatography (HPLC). The purity of the peptides was more than 95%. These peptides were stored at −80°C after lyophilization in DMSO (Sigma) and diluted in endotoxin free PBS (GIBCO, New York, NY, United States) when used.

### Binding Ability of LppZ Peptides to HLA-A2 Molecules

T2 cells were purchased from ATCC and were maintained in Iscove’s Modified Dulbecco’s Media (IMDM) (GIBCO) supplemented with 10% fetal bovine serum (FBS) (Millipore, Burlington, MA, United States), 100 units/mL penicillin (GIBCO) and 100 μg/mL streptomycin (GIBCO). T2 cells were harvested and washed with IMDM serum-free medium. The cell concentration was adjusted to 1 × 10^6^ cells/mL, and were then stimulated with 10 μg/mL peptides in the presence of 3 μg/mL beta 2 microglobulin (β2-M) (Sigma-Aldrich, St. Louis, MO, United States) and incubated in 5% CO_2_ incubator for 4 h at 37°C. The expression of HLA-A2 molecules on T2 cells was determined by PE-conjugated mouse anti-human HLA-A2 (clone: BB7.2) (Abcam, United Kingdom), and cells were then incubated for 30 min in the dark at 4°C. After wash and resuspended in PBS, T2 cells were acquired by flow cytometer (BD FACSCanto II, San Jose, CA, United States). Binding ability of predicted HLA-A2-restricted LppZ peptides to T2 cells were determined based on the mean fluorescence intensity (MFI) values of HLA-A2 molecule expression. Peptide L235 was considered as a positive control, PBS (no antigenic peptide) as a negative control.

### Binding Affinity and Binding Stability Assay of HLA-A2-Restricted LppZ Peptides

T2 cells were stimulated with HLA-A2-restricted LppZ peptides in different concentrations (0, 0.4, 2, 10 μg/mL) in the presence of β2-M for 4 h. The expression levels of HLA-A2 molecules on T2 cells were determined by flow cytometry as mentioned above. The binding affinity of LppZ-derived peptides to HLA-A2 molecules was defined by the concentration at which it displayed 50% of the maximum MFI (MFI_max_) of labeled HLA-A2 molecules.

For binding stability assay, T2 cells were stimulated LppZ-derived peptides at optimal concentration (10 μg/mL) in the presence of β2-M for 4 h. The MFIs of HLA-A2 molecule expression on T2 cells were detected at 0, 2, 4, and 6 h separately after 4 h stimulation. The binding stability of LppZ-derived peptides to HLA-A2 molecules was defined by the difference between MFI_0hr_ and MFI_6hr_ (Δ MFI = MFI_0 h_ - MFI_6 h_).

### Peripheral Blood Mononuclear Cell Isolation

Whole blood (10 mL) was collected in tubes containing ethylene diamine tetraacetic acid (EDTA). Peripheral blood mononuclear cells (PBMCs) were isolated by Ficoll-hypaque density gradient centrifugation with Lymphoprep^TM^ solution (AXIS-SHIELD Poc AS, Oslo, Norway) according to the manufacturer’s recommendation. The mononuclear cell layer was carefully transferred to a new 15 mL conical tube and washed twice with RPMI 1640 medium (GIBCO) by centrifuging at 486 × *g* for 10 min at room temperature. PBMCs were re-suspended at a concentration of 2.5 × 10^6^/mL in RPMI 1640 culture medium containing 10% FBS, 100 units/mL penicillin and 100 μg/mL streptomycin.

### HLA-A2 Haplotype Determination

Add 1.0 ml of lysing solution (BD) to a tube containing 100 μl anti-coagulant whole blood. Gently vortex tube immediately, and incubate at room temperature in the dark for 15 min. Centrifuge at 200 × *g* for 5 min, and carefully aspirate supernatent. Add 1.0 ml FACS buffer (PBS containing 2% FBS), centrifuge again and carefully aspirate supernatent. The pellet was resuspended in FACS buffer containing PE-conjugated mouse anti-human HLA-A2 monoclonal antibody, and incubate in the dark for 40 min at 4°C. Cells were then washed and resuspended in PBS. The samples were further analyzed by flow cytometer (BD FACSCanto II). Only HLA-A2 positive individuals were recruited in this study.

### Interferon Gamma (IFN-γ) Release Assay (IGRA)

Antigen-specific IFN-γ releasing levels were determined by using an enzyme-linked immunospot (ELISpot) assay according to the manufacturer’s instructions (U-CyTech, Utrecht, Netherlands). Briefly, 96-well PVDF plates (Millipore) were coated with anti-human IFN-γ coating antibody overnight at 4°C. The wells were blocked for 1 h at 37°C. 2.5 × 10^5^ PBMCs in 100 μL RPMI 1640 culture medium were inoculated in each well and stimulated with HLA-A2-restricted LppZ peptides, E6p or C10p pools (2 μg/mL per peptide). RPMI 1640 culture medium served as a negative control and 2.5 μg/mL phytohemagglutinin (PHA) (Sigma-Aldrich) was used as a positive control. After 20 h incubation at 37°C, the plates were incubated with biotin-labeled detection antibody at 37°C for 1 h and subsequently HRP-conjugated streptavidin working solution for another 1 h. AEC substrate solution was added to each well for 30 min in the dark at room temperature. Color development was stopped by thoroughly rinsing both sides of the PVDF membrane with demineralized water. The plates were dried in the dark at room temperature. The spots were counted by C.T.L. ImmunoSpot^®^ S6 Ultra Analyzer (Cellular Technology Limited, Shaker Heights, OH, United States). The number of antigen-specific IFN-γ-producing cells was calculated based on spot-forming units (SFUs) per 2.5 × 10^5^ PBMCs after deducting the background SFUs detected by the paired negative control wells.

### Detection of Intracellular Cytokines

T cell functionality was determined by intracellular cytokine staining (ICS) after antigen stimulation as described in a previous study (Xiao et al., 2016). Freshly isolated PBMCs were inoculated in the 96-well U-bottom plates (Corning, PA, United States) at 1 × 10^6^ /well. Cells were stimulated with E6p and C10p peptides pool (E6C10p), HLA-A2-restricted LppZp7/p10 peptides pool (2 μg/mL per peptide), and incubated in 5% CO_2_ incubator for 20 h at 37°C. GolgiStop (BD Bioscience) was added 4 hrs before the end of *ex vivo* stimulation. For surface staining, cells were labeled with Pacific Blue-conjugated mouse anti-human CD3, PE-Cy7-conjugated mouse anti-human CD8, and FITC-conjugated mouse anti-human CD45RO (all from BD Bioscience) for 40 min in the dark at 4°C. After washing with FACS buffer, cells were fixed with Cytofix/Cytoperm solution and permeated with Fix/Perm working solution (BD Biosciences). Cells were then stained with PerCP-Cy5.5-conjugated mouse anti-human IFN-γ (BD Biosciences), APC-conjugated mouse anti-human IL-2 (BioLegend, San Diego, CA, United States) and PE-conjugated mouse anti-human TNF-α (eBioscience, San Diego, CA, United States) antibodies for 40 min in the dark at 4°C. After wash and resuspended in PBS, cells were acquired with flow cytometer (BD FACSCanto II) in 2 h. Data were analyzed by using FlowJo software 10 (FlowJo LLC, Treestar Inc., Ashland, OR, United States), and the doublet events were excluded by FSC-A/FSC-H gating.

### Statistical Analysis

All the data are shown as mean ± SEM. Statistical analyses were performed by using GraphPad Prism 7 software (Graphpad software Inc., La Jolla, CA, United States). Statistical differences were assessed by the unpaired *t*-test for the data with Gaussian distribution and by the Mann–Whitney test for those with non-Gaussian distribution.

## Results

### Study Subjects

HLA-A2 positive (HLA-A2^+^) ATB patients (*N* = 15) and HCs (*N* = 20) were involved in this study (Table 1). HLA-A2^+^ ATB patients (age: 44 ± 16.25; female/male: 7/8) included new onset patients (*N* = 11) and the retreated patients (*N* = 4). Ten (66.66%) were sputum smear positive, and eight (53.33%) were diagnosed with pulmonary cavitation (Table 2).

**Table 1:** Epidemiological information of HLA-A2^+^ population.

	ATB	HC
N	15	20
Gender(Male/Female)	7/8	13/7
Age(Mean ± SEM)	44.95 ± 16.19	30.75 ± 6.28
Sputum smear (+/−)	9/6	
Cavity (+/−)	8/7	

**Table 2:** Clinical information of HLA-A2^+^ TB patients.

Number	Gender	Age	Anti-tuberculosis treatment	Therapeutic drugs	Initial treatment/Re-treatment	Diagnosis	Merger disease	Pulmonary cavity	Sputum smear
TBp1	F	64	13M	HREZ	Initial treatment	Secondary tuberculosis	Leukopenia	−	**+**
TBp2	M	74	17M	HRZM	Re-treatment	Pulmonary tuberculosis	Hyperbilirubinemia	−	**+**
TBp3	M	63	−	HRZM	Initial treatment	Renal tuberculosis	−	**+**	−
TBp4	F	64	−	HRZM	Initial treatment	Tuberculosis	Renal dysfunction	**+**	−
TBp5	M	39	13M	HRZM	Re-treatment	Mediastinal lymph node tuberculosis	−	−	−
TBp6	M	66	1M	HRZM	Re-treatment	Secondary tuberculosis	Diabetes	−	**+**
TBp7	F	30	3M	HRZM	Initial treatment	Secondary tuberculosis	−	**+**	**+**
TBp8	M	35	12M	HRZM	Initial treatment	Mediastinal lymph node tuberculosis	Fatty liver disease	**+**	**+**
TBp9	F	24	2M	HRZM	Initial treatment	Secondary tuberculosis	Anemia	**+**	**+**
TBp10	M	23	16M	HRZM	Initial treatment	Secondary tuberculosis	Hyperuricemia	−	−
TBp11	M	59	2M	HRZM	Initial treatment	Tuberculosis	−	**+**	**+**
TBp12	F	32	48M	HRZM	Initial treatment	Secondary tuberculosis	−	−	**+**
TBp13	M	46	3M	HRZM	Initial treatment	Secondary tuberculosis	Low tension hypoxia	**+**	**+**
TBp14	F	35	19M	HRZM	Re-treatment	Pulmonary tuberculosis	−	−	**+**
TBp15	M	20	1M	HRZM	Initial treatment	Secondary tuberculosis	Tracheal tuberculosis	**+**	−

*H, Isoniazid; R, Rifampicin; E, Ethambutol; Z, Pyrazinamide; L, Levofloxacin; M, Moxifloxacin.*

### Prediction of HLA-A2-Restricted LppZ Peptides by Bioinformatics

LppZ is a 38 kDa protein with 373 amino acid residues^1^. HLA-A2-restricted peptides were predicted on the website http://www-bimas.cit.nih.gov/molbio/hla_bind. Ten LppZ peptides were screened out (Table 3) with the predicted binding affinity score. The higher the score was, the higher the affinity to HLA-A2 molecule was theoretically.

**Table 3:** Predicted HLA-A2-restricted peptides derived from LppZ sequence.

Peptide	Position	Amino acid subsequence	Bioinformatic score
LppZp1	p350–358	KLDDVVFPL	1635.753
LppZp2	p10–18	GLAALCAAV	69.552
LppZp3	p291–299	NLINTKLTV	69.552
LppZp4	p137–146	RLMYAYIST	41.359
LppZp5	p270–276	WTWPDKPGV	36.882
LppZp6	p204–212	SLAGKVLRI	23.995
LppZp7	p197–205	ALAADPQSL	21.362
LppZp8	p178–186	LIFTSPTTL	18.476
LppZp9	p282–290	AAMDGTVLV	14.654
LppZp10	p69–77	VMQGCLEST	10.311
L235		FLPDHINIV	1236.9

### Determination of Binding Ability of Predicted HLA-A2-Restricted LppZ Peptides to HLA-A2 Molecules

**FIGURE 1:**
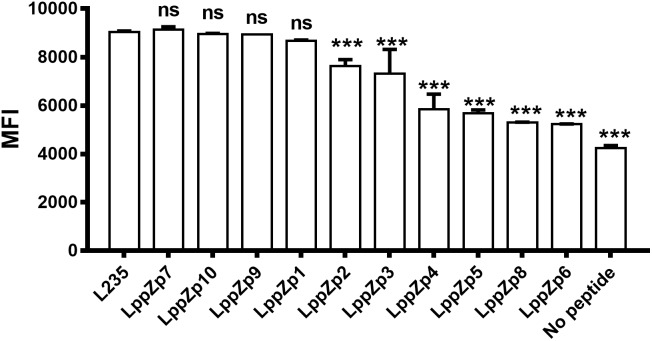
Binding capacity of HLA-A2-restricted LppZ peptides to T2 cell. The *P-value* was calculated using the Mann–Whitney test. ^∗∗∗^*p* ≤ 0.001. L235, positive control; No peptide, negative control.

Ten predicted HLA-A2-restricted LppZ peptides were subjected to a binding ability assay. T2 cells were stimulated with individual peptide at 10 μg/mL *in vitro*, and the expression level of HLA-A2 molecules on T2 cells was determined. L235 is a peptide derived from OVA66, which is already proved to display high ability to the HLA-A2 molecules on T2 cell surface (Jin et al., 2005). No peptide presented the background of HLA-A2 expression on T2 cells without peptide stimulation. The results showed that among ten predicted peptides, LppZp1, LppZp7, LppZp9, and LppZp10 stabilized HLA-A2 expression on T2 cells after 4-h incubation with MFI values similar to the positive peptide control L235 (Figure 1), whereas incubation of T2 cells with the resting predicted peptides displayed significantly lower MFI values of HLA-A2 expression than that of L235 (*p* = 0.0001). Therefore, LppZp1, LppZp7, LppZp9, and LppZp10 exhibit strong binding ability to HLA-A2 molecules among predicted LppZ peptides.

### Binding Affinity of HLA-A2-Restricted LppZ Peptides to HLA-A2

**FIGURE 2:**
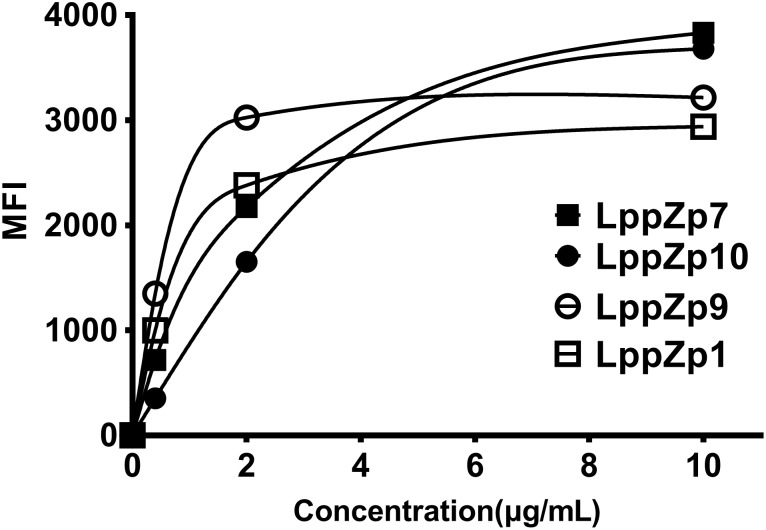
Binding assay of LppZ-derived peptides to HLA-A2 molecules.

In order to further determine the binding affinity of LppZp1, LppZp7, LppZp9, and LppZp10 to HLA-A2 molecules more quantitatively, peptides at different concentrations (0, 0.4, 2, and 10 μg/mL) were incubated with T2 cells in the presence of β2-M and the corresponding MFI values were detected. The results showed that along with the increase in peptide concentration, the expression levels of HLA-A2 molecules on T2 cells augmented in parallel (Figure 2). However, four LppZ peptides displayed different increasing curves. At low concentration level (2 μg/mL), LppZp9 (3025) and LppZp1 (2380) exerted higher MFI values than LppZp7 (2172) and LppZp10 (1652), whereas LppZp7 (3831) and LppZp10 (3679.5) exhibited dramatically increased affinities to HLA-A2 molecules than LppZp9 (3216) and LppZp1 (2938) at the concentration of 10 μg/mL. Both LppZp9 and LppZp1 have reached the MFI platform at 2 μg/mL. Peptide concentrations of LppZp1 (0.65 μg/mL) and LppZp9 (0.49 μg/mL) at 50% MFI_max_ were lower LppZp10 (2.27 μg/mL) and LppZp7 (1.54 μg/mL).

### Binding Stability of HLA-A2-Restricted LppZ Peptides to HLA-A2

**FIGURE 3:**
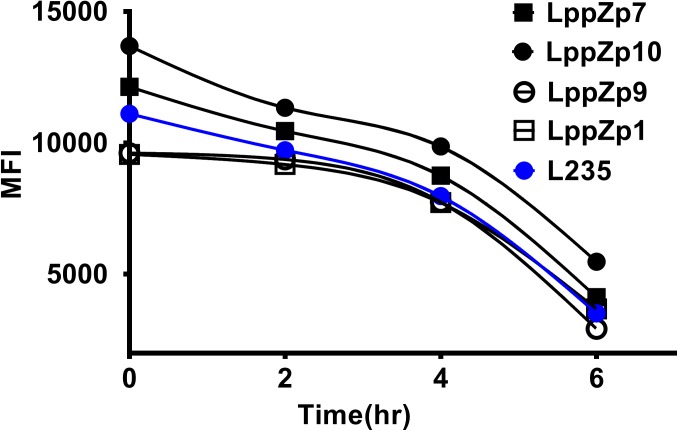
Binding stability assay of LppZ-derived peptides to HLA-A2 molecules.

Peptide binding stability is another criteria to define the binding affinity of specific peptides. The assay was performed through determining the HLA-A2 expression levels at different time points after the incubation with T2 cells. It was shown that the MFI values of HLA-A2 molecules on T2 cells displayed a dramatic decrease along with the duration of incubation times both in LppZ peptides and L235 positive peptide. Four LppZ peptides could be subgrouped into two according to the HLA-A2 expression patterns: LppZp7 and LppZp10 peptides displayed higher binding stability than L235 peptides whereas binding stability of LppZp1 and LppZp9 peptides were lower (Figure 3).

Considering the results from T2 binding affinity and stability assays, LppZp7 and LppZp10 were classified as peptides binding better to HLA-A2 molecule. We thus defined LppZp7 and LppZp10 as candidate peptides for further clinical evaluation of cellular immune responses in TB patients.

### LppZp7 and LppZp10 Peptides Exert Strong Immunoreactivity in HLA-A2^+^ ATB Patients

**FIGURE 4:**
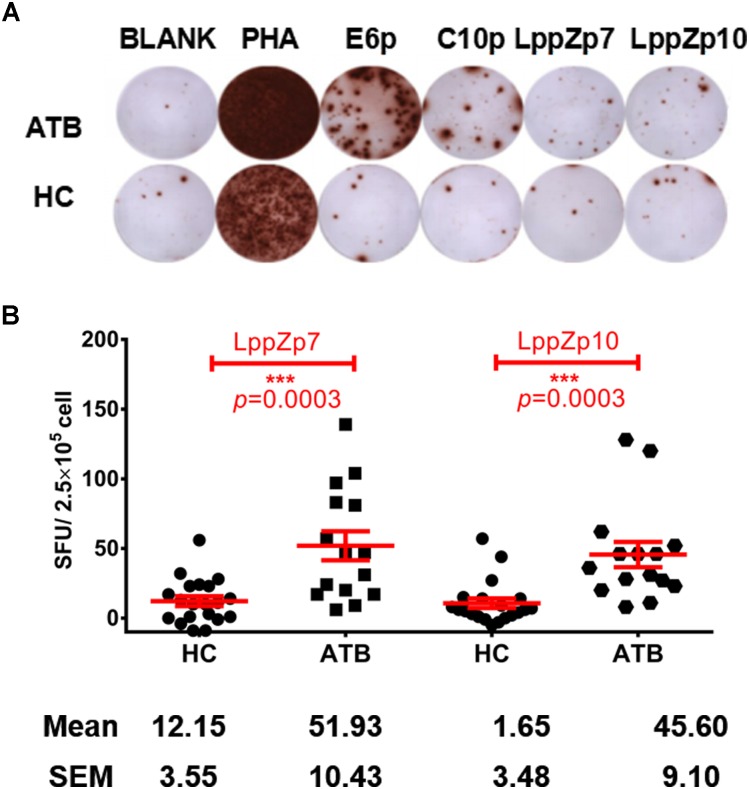
LppZp7 and LppZp10-specific IFN-γ releasing levels in HLA-A2^+^ population. **(A)** A representative of the IFN-γ ELISpot assay. PBMCs from one ATB patient and one HC were stimulated with ESAT-6 peptide pool (E6p), CFP-10 peptide pool (C10p), LppZp7 or LppZp10, and PHA (as the positive control). No stimulant blank was used as blank control. **(B)** The numbers of IFN-γ secreting cells (per 250,000 PBMCs) from ATB patients (*N* = 15) and HCs (*N* = 20) following stimulation with indicated LppZ peptides determined by ELISpot. The *P-value* was calculated using the Mann-Whitney test. ^∗∗∗^*p* ≤ 0.001.

Our previous work has demonstrated that LppZ was an immune dominant antigens inducing strong immune responses in ATB patients and LTBI cohorts (Xiao et al., 2017). Whether LppZp7 and LppZp10, with strong and stable binding ability to HLA-A2 molecule defined in the above mentioned *in vitro* T2 binding screening assays, possess the similar ability to trigger cellular immune response was further investigated. IFN-γ ELISpot assay was adapted to define the immunoreactivity of these two LppZ-derived peptides in ATB patients as well as in HCs. As shown in Figure 4, both LppZp7 and LppZp10 have triggered more IFN-γ producing cells in ATB patients (51.93 ± 10.43 and 45.60 ± 9.10 SFUs/2.5 × 10^5^ PBMCs, respectively) than HCs (12.15 ± 3.55 and 1.65 ± 3.48 SFUs/2.5 × 10^5^ PBMCs, respectively) with statistical significance (*p* = 0.0003) (Figure 4B). Furthermore, IFN-γ secretion induced by HLA-A2-restricted peptides pool was abolished by addition of BB7.2 mAb and CD8 depletion (Supplementary Figure S1).

**FIGURE 5:**
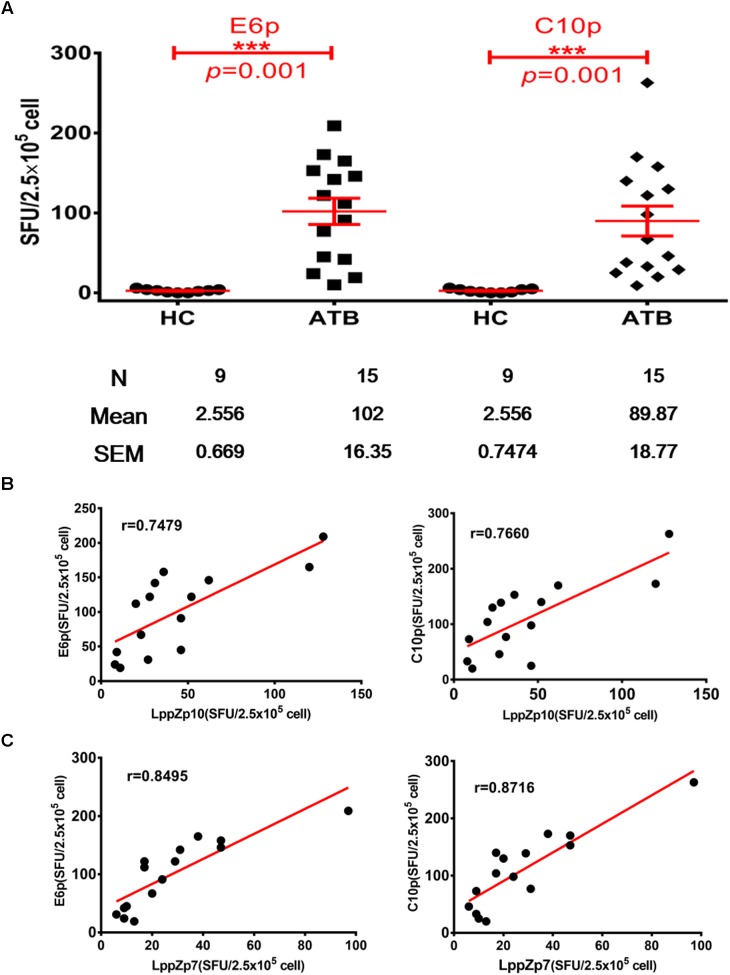
Correlations between HLA-A2-restricted LppZ peptide-specific and E6p or C10p specific IFN-g release. **(A)** Numbers of E6p- or C10p-specific IFN-γ producing cells from HLA-A2^+^ ATB patients (*N* = 15) and HC (*N* = 20). The *P-value* was calculated using the Mann–Whitney test. **(B)** Correlation analysis between the LppZp10-specific IFN-γ releasing level and that to E6p (left, *r* = 0.7479, *p* = 0.0039) or C10p (right, *r* = 0.7660, *p* = 0.0022) in ATB patients (*N* = 15). **(C)** Correlation analysis between the LppZp7-specific IFN-γ releasing level and that E6p (left, *r* = 0.8495, *p* = 0.0001) or C10p (right, *r* = 0.8716, *p* = 0.0001) in ATB patients (*N* = 15). The correlation coefficient *r* and the *P-value* were calculated using the Mann–Whitney test. ^∗∗∗^*p* ≤ 0.001.

**FIGURE 6:**
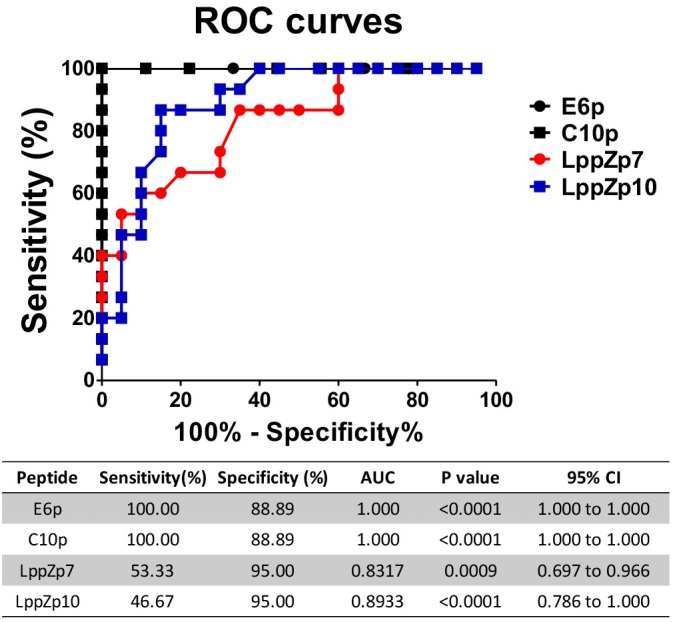
Receiver operating characteristic curve analyses of antigen specific IFN-γ producing cells between HC individuals and TB patients. The *P*-values were calculated using Mann–Whitney test. AUCs were listed in the table.

ESAT-6 and CFP-10 are two well-known immune dominant antigens specifically existing in *M.tb*. They are subjected to immunological diagnosis for *M.tb* infection. We further compared the response levels between LppZp7 or LppZp10 peptides and ESAT-6 (E6p) or CFP-10 (C10p) peptide pools in ATB patients. Our results showed that the immune responses to E6p and C10p in ATB patients (102 ± 16.35 and 89.87 ± 18.77 SFUs/2.5 × 10^5^ PBMCs, respectively) were significantly higher than those in HC group (2.556 ± 0.669 and 2.556 ± 0.747 SFUs/2.5 × 10^5^ PBMCs, respectively) (*p* = 0.001) (Figure 5A). More importantly, there existed significantly positive correlations between LppZp10 and E6p/C10p (*r* > 0.7) (Figure 5B) as well as between LppZp7 and E6p/C10p (*r* > 0.8) (Figure 5C) in ATB population. Since ATB patients responded to LppZ-derived HLA-A2-restricted peptides in a consistent manner as to E6p or C10p, receiver operating characteristic curve (ROC curve) analyses were performed to further evaluate the diagnostic potential of LppZ peptides-specific IFN-γ production in ATB subjects. It was demonstrated that LppZ peptides-induced cellular responses could also discriminated ATB patients from HCs with the AUC value of 0.8317∼0.8933. The sensitivity of LppZp7 and LppZp10 were 53.33 and 46.67%, respectively while the specificity was 95.00% (Figure 6, red dot and blue square). Those peptides have exhibited higher specificity than E6p or C10p (Figure 6, black dot and square), although the sensitivity was lower. These results, however, indicate that HLA-A2-restricted LppZp7 and LppZp10 peptides display apparent immunoreactivity equivalent to E6p and C10p in ATB patients.

### LppZp7 and LppZp10 Induce Antigen-Specific Th1-like T Cells Responses in HLA-A2^+^ ATB Patients

**FIGURE 7:**
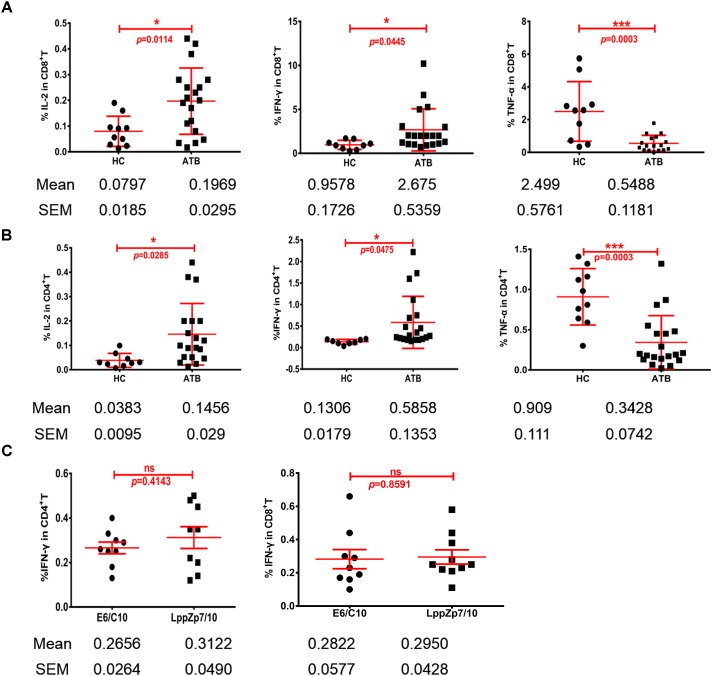
HLA-A2-restricted LppZ peptides induce antigen specific responses in both CD8^+^ and CD4^+^ T cells. **(A)** LppZp7/10-specific IFN-γ and IL-2 release in CD8^+^ T cells from ATB patients and HCs. **(B)** LppZp7/10-specific production of IFN-γ and IL-2 in CD4^+^ T cell from ATB patients and HCs. **(C)** Comparison of IFN-γ releasing levels between E6p/C10p and LppZp7/10 stimulation in peripheral CD4^+^ and CD8^+^ T cells from ATB patients. ^∗^*p* ≤ 0.05; ^∗∗∗^*p* ≤ 0.001.

Cytokine production is one of the key signatures for T cell functionality. The ability of HLA-A2-restricted LppZ peptides to trigger CD8^+^ T cell response was further studied through detecting the production of Th1-type cytokines. PBMCs from HLA-A2^+^ ATB patients were stimulated *ex vivo* with peptide LppZp7, LppZp10 or the combination of LppZp7 and LppZp10 peptides (LppZp7/p10) and Th1-type cytokines were detected by ICS. RPMI1640 culture medium served as unstimulation control which defined the background cytokine release level (data not shown). The results showed that although the numbers of cytokine-secreting CD8^+^ T cells stimulated with two peptide alone were few (data not shown), stimulation of LppZp7/p10 induced more frequencies of IL-2^+^CD8^+^ T cells (0.197 ± 0.129%) and IFN-γ^+^CD8^+^ T cells (2.67 ± 2.39%) in ATB patients than those in HCs (IL-2^+^CD8^+^ T cells: 0.079 ± 0.058%, *p* = 0.0114; IFN-γ^+^CD8^+^ T cells: 0.957 ± 0.517%, *p* = 0.0445). However, the frequency of TNF-α secreting CD8^+^ T cells (0.548 ± 0.486%) in ATB group was significantly lower than that in HCs (2.498 ± 1.82%) (*p* = 0.0003) (Figure 7A). Nevertheless, HLA-A2-restricted LppZp7/p10 peptides were able to induce apparent CD8^+^ T cell immune responses in ATB patients.

More interestingly, the generation of Th1-type cytokine was also detectable in CD4^+^ T cells from HLA-A2^+^ ATB patients. The cytokine release pattern in CD4^+^T cells was quite similar with that in CD8^+^T cells. Significantly higher frequencies of IL-2^+^CD4^+^ T cells (0.145 ± 0.126%) and IFN-γ^+^CD4^+^ T cells (0.58 ± 0.60%) were observed in ATB patients when compared with those in HC group (IL-2^+^CD4^+^ T cells: 0.039 ± 0.030%, *p* = 0.0285; IFN-γ^+^CD4^+^ T cells: 0.134 ± 0.056%, *p* = 0.0475) (Figure 7B). We have also compared the secretion levels of IFN-γ in CD4^+^ and CD8^+^ T cells upon either LppZp7/10 mixture or E6p/C10p stimulation in HLA-A2^+^ ATB patients. There were no difference between two types of antigenic peptide stimulation (Figure 7C).

These results demonstrate that LppZp7 and LppZp10 peptides induce not only CD8^+^ T cell responses, but also CD4^+^ T cell responses.

## Discussion

Although *M.tb* specific ESAT-6 and CFP-10 are recognized as the most immunogenic antigens that are successfully used in the immunodiagnosis of TB (Lewinsohn et al., 2010), their application in vaccine development is less promising due to strong inflammatory reactions especially in patients receiving immunosuppressive agents or lymphocytopenia patients (Kobashi et al., 2008). Screening new mycobacterial antigens is thus still of great significance in both the development of TB vaccines and new immunodiagnostic tools, as well as in the potential application of immunotherapy against TB.

Th1 cytokines release CD4 T are considered as main factor against *M.tb* infection, while recent study showed that CD8 T cells mediated immune responses can also protect mouse from H37Rv challenge (Hu et al., 2017). HLA-A2-restricted peptides are usually considered to induce cytotoxic activity in CD8^+^ T lymphocytes. Furthermore, peptide vaccines are one of the most promising strategies for the prevention and treatment of tumors (Sahin et al., 2017; Tsuchiya et al., 2017). The predicted candidate antigenic peptides are further determined by experimental investigation and clinical trials against tumors (Chheda et al., 2018). LppZ is one of the proteins identified by our group with high immunogenicity and antigenicity (Xiao et al., 2016, 2017). Our unpublished data also indicated that LppZ vaccination is protective in mouse models (under revision manuscript). Our present study might provide another choice represented by LppZ peptides that are dominant mycobacterial antigens with good immunogenicity while less inflammatory responses, thus making LppZ peptides new candidate peptides in the application of immunotherapy for the refractory TB in the future.

In this study, we have successfully mapped two HLA-A2-restricted LppZ epitopes (LppZp7 and LppZp10) by bioinformatics and affinity binding assay. In fact, computational prediction highlighted LppZp1 with the highest binding score. However, results from T2 binding affinity assay indicated that LppZp1 displayed similar binding ability to HLA-A2 molecules as LppZp7 and LppZp10, but its binding stability was less than other two LppZ peptides. Multiple molecules are involved in HLA class I-restricted antigen presentation, among which transporters associated with antigen processing (TAPs) are responsible for the transportation of cytosol peptides into endoplasmic reticulum. A recent investigation on Gaussian process (GP) model has shown that the P1, P2, P3, and P9 residues are pivotal positions that dominate TAP-peptide recognition (Ren et al., 2011) while P2 and P9 residues are anchoring residues for binding to HLA class I molecules. We then evaluated the hydrophobicity and hydrophilicity of amino acid residues on these sites (Zhu et al., 2016). LppZp7 possesses hydrophobic residues (A_1_L_2_A_3_ and L_9_) at all anchor positions while LppZp1 holds 3 (K_1_L_2_ and L_9_) and LppZp10 (V_1_M_2_) has only 2 hydrophobic residues, respectively. Surprisingly, more hydrophilic amino acid residues were found in LppZp7 (D_5_ and Q_7_S_8_) and LppZp10 (G_4_C_5_ and S_8_) than in LppZp1 (D_4_). Therefore, the similarity in anchoring residues with HLA-A2 molecules is not sufficient enough for the precision prediction of peptides. While anchoring residues direct the binding of the peptides to the corresponding HLA molecules, the remaining residues might affect the molecular interaction between peptides and other molecules involved in antigen presentation, which finally determine the binding properties of peptides to HLA molecules and the presentation on cell surface. Therefore, computational prediction combined with T2 cell-based experimental screening remains the reliable strategy to determine the antigen peptide. Similar results have also been reported in other studies (Mizukoshi et al., 2011; Bai et al., 2018).

Whether LppZp7 and LppZp10 possess the ability to trigger immunoreactivity in TB patients was further investigated. LppZp7 and LppZp10-specific IGRA assays were performed in ATB patients and HCs. Consistent with the results from LppZ protein (Xiao et al., 2017), both LppZp7 and LppZp10 peptides induced more dramatic cellular immune responses in HLA-A2^+^ ATB patients than in HC counterparts (Figure 4). What is more, the responsiveness to both LppZp7 and LppZp10 displays significantly positive correlations to E6p/C10p in ATB group, demonstrating that the LppZ-derived peptides bear comparable immunogenicity to ESAT-6 and CFP-10, which confirmed the immunoreactivity of the whole protein in our previous study (Xiao et al., 2017).

LppZp7 and LppZp10 are HLA class I-restricted peptides. They were expected to induce cytokine production in CD8^+^ T cells. Although single peptide could hardly generate the secretion of Th1-type cytokines in CD8^+^ T cells (data not shown), *ex vivo* combinational treatment of LppZp7 and LppZp10 led to more IFN-γ^+^ and IL-2^+^ release CD8^+^ T cells in ATB patients than those in HC group. As a typical chronic infectious disease characterized as delayed type hypersensitivity responses, persistent mycobacterial antigens can stimulate T cells to release cytokines such as IFN-γ, IL-2 and TNF-α continuously. IFN-γ is one of the key signature cytokines for CD8^+^ T cell functionality. Mycobacterial antigen-specific IFN-γ level is considered to be closely related to *M.tb* infection state. Hinks et al. (2012) found that the number of specific IFN-γ cells upon the stimulation of antigens from *M.tb* RD1 region was related to bacilli status in patients. Studies from Park (Park and Shim, 2017) and our group (Wu et al., 2017) indicated that the number of antigen-specific IFN-γ producing cells decreased along with the anti-TB treatment and the subsequent decrease in bacilli burden. Out of our expectation, LppZ peptide-induced TNF-α secretion was significantly lower in CD8^+^ T cells from ATB patients when compared to HC group. It is believed that protection and pathology in TB are modulated to certain extent by TNF-α. The decreased secretion level of TNF-α in ATB population to some extent represents the pathological status of TB patients (Dorhoi and Kaufmann, 2014). Besides, loss of TNF-α might also due to the T cell exhaustion in TB patients (Jayaraman et al., 2016). It has been reported that the levels of TNF-α are lower in the pathogenesis of bronchitis and sepsis as well (Wada et al., 1991; Marchant et al., 1996). Increasing antigen-specific TNF-α response might be used as one of the criteria for successful vaccination in TB vaccine development (Maggioli et al., 2016).

Interestingly, upon LppZ peptides *ex vivo* stimulation, not only cytokine production in CD8^+^ T cell, but also in CD4^+^ T cells was observed. LppZp7 and LppZp10 are HLA-A2-restricted peptides whereas antigens that trigger specific CD4^+^T cell responses are often MHC class II molecules restricted. One possibility lies in the fact that LppZp7 and LppZp10 can bind to MHC class II molecules as well with weak ability. However, blockade with BB7.2 mAb revoked cytokine release (Supplementary Figure S1). Thus, the most possibility is that cytokines produced by activated CD8^+^ T cells can function on CD4^+^ T cells, which reflects by-stand effects of HLA class I-restricted peptides. It is reported that T cell vaccine-mediated immune protection on autoimmune encephalomyelitis model is orchestrated by CD4^+^ and CD8^+^ T cells (Fiebiger et al., 2015). Although the exact mechanism needs to be clarified further, our results thus suggest that HLA class I-restricted peptides can still effectively trigger the activation of CD4^+^ T cells, which is expected to play synergistic roles in anti-TB immunity. The roles of CD8^+^ T cells have been reported as well (Hu et al., 2017).

In summary, we have identified HLA-A2-restricted LppZp7 and LppZp10 peptides with good binding capacity and binding stability. They also exert higher immunoreactivity in ATB patients with antigen-specific CD8^+^ and CD4^+^ T cell responses. With the ability of LppZp7 and LppZp10 to trigger strong immune responses against TB, their potential value in vaccine development and diagnosis are worthy of further exploration.

## Ethics Statement

This study was carried out in accordance with the recommendations of the Ethical Committee of Shanghai Jiao Tong University School of Medicine with written informed consent from all subjects. All subjects gave written informed consent in accordance with the Declaration of Helsinki. The protocol was approved by the Ethical Committee of Shanghai Jiao Tong University School of Medicine.

## Author Contributions

YW and YC designed the experiments. Y-yL, WS, and YC conducted the experiments. YW, YC, XZ, G-pZ, Y-yL, WS, and SX analyzed the data. WS and X-wG collected the samples and clinical data. LX, PJ, and SW contributed reagents, materials, and analysis tools. Y-yL, XZ, YC, and YW wrote the manuscript.

## Conflict of Interest Statement

The authors declare that the research was conducted in the absence of any commercial or financial relationships that could be construed as a potential conflict of interest.

## Footnotes

1 http://svitsrv8.epfl.ch/tuberculist
