# Supplementary material for: Identification of HLA-A2-Restricted Mycobacterial Lipoprotein Z Peptides Recognized by T CellsFrom Patients With ActiveTuberculosis Infection
Source: Front Microbiol. 2018 Dec 21;9:3131. doi: 10.3389/fmicb.2018.03131 (PMC6308912; doi:10.3389/fmicb.2018.03131)
Supplement: Supplementary file 1 [file Presentation_1.pdf]

## Supplementary Material

# Identification of HLA-A2-Restricted Mycobacterial Lipoprotein Z Peptides Recognized by T Cells From Patients With Active in Tuberculosis Infection

**Yuan-yong Liu[1,2†], Wei Sha[3†], Shiqiang Xu[2], Xu-wei Gui[3], Liliang Xia[2], Ping Ji[2], Shujun Wang[2], Guo-ping Zhao[4], Xiao Zhang[1*], Yingying Chen[2*] and Ying Wang[2,4]**

*** Correspondence:** Corresponding Authors: Prof. Xiao Zhang, zhangxiao@cust.edu.cn; PhD.Yingying Chen, yingying.chen@shsmu.edu.cn

## Supplementary Figures

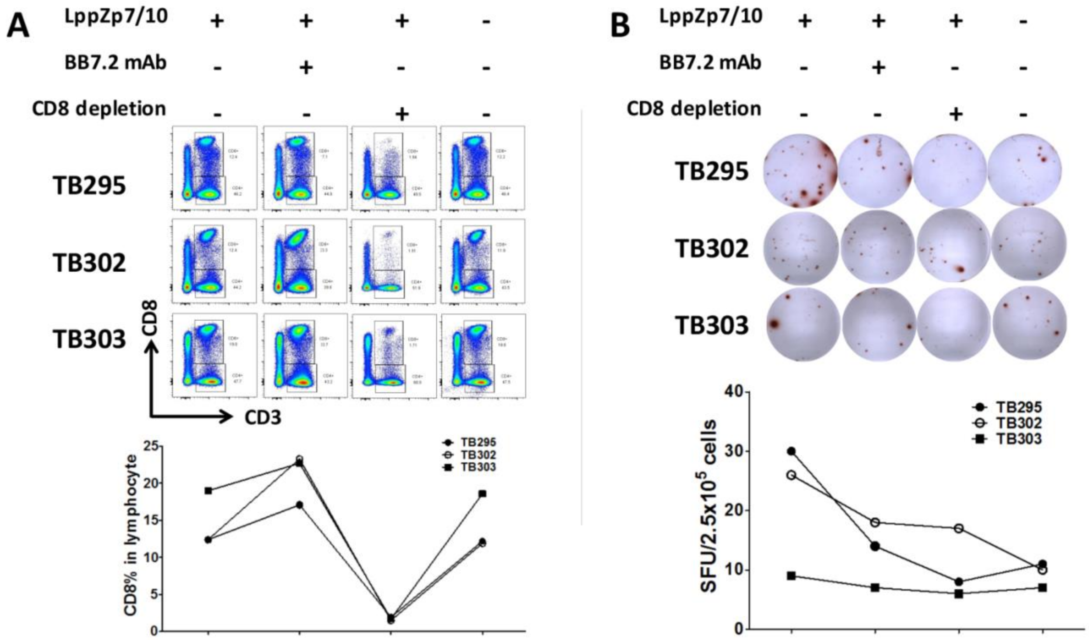


**Supplementary Figure 1.** CD8 depletion and HLA-A2 blockade could abolish the IFN-γ secretion triggered by LppZp7/10 pool in TB patients. A) Frequency of $CD8^{+}$T cells upon BB7.2 mAb blockade (Abcam) or CD8 depletion. B) LppZp7/10 pool-induced IFN-γ releasing cells decreased in the presence of BB7.2 mAb or upon CD8 depletion. CD8 T cells in PBMC derived from TB patients peripheal blood were depleted by human CD8 MicroBeads (Miltenyi Biotec).
